# Complete Genome Sequences of Four Phages of the Horse Chestnut Phyllosphere

**DOI:** 10.1128/MRA.00821-21

**Published:** 2021-11-04

**Authors:** Greg P. Krukonis, Sam J. Roth, Véronique A. Delesalle

**Affiliations:** a Department of Biology, Angelo State University, San Angelo, Texas, USA; b Department of Biology, Gettysburg College, Gettysburg, Pennsylvania, USA; Portland State University

## Abstract

Bacteriophages play important roles in determining bacterial communities, including plant microbiota. Here, we describe four lytic phages, three *Siphoviridae* and one *Podoviridae*, isolated from four different bacterial species found on the leaves of horse chestnut trees. Their double-stranded DNA (dsDNA) genomes range from 39,095 to 46,062 bp and contain 51 to 70 genes.

## ANNOUNCEMENT

To understand the roles bacteriophages play in the phyllosphere, phages found on the leaves of horse chestnut trees (Aesculus hippocastanum; Sapindaceae) in Angel and Greyhound Meadow, Oxford, UK, were isolated on bacterial strains, themselves isolated from these leaves (1–5). The bacterial isolates were assigned to a genus and, if possible, species based on sequencing of approximately 800 bp of the 16S rRNA region and the top BLAST hit associated with a sequence (E value, <10^−10^) (1). Here, we describe four of these phages, each isolated on a different bacterial species (Table 1).

**TABLE 1:** Isolation and genome characteristics for phages AH01, AH02, AH03, and AH05^*a*^

Phage name	Isolation host (median %GC)	Yr of isolation	No. of reads	Coverage (×)	Genome ends	Genome size (bp)	%GC	Protein genes (% with function)	BLASTn query results		
									Best match (GenBank accession no.)	Coverage (%)	Identity (%)
AH01	*Pantoea* sp. (55.0)	2014	498,698	1,624	Headful packaging	46,062	52.2	69 (40.6)	Salmonella phage St162 (MF158037.1)	59	74.13
AH02	Pseudomonas koreensis (60.2)	2014	339,084	1,301	Circularly permuted	39,095	54.9	70 (40.0)	Pseudomonas phage MR15 (MT104475.1)	2	81.76
AH03	Erwinia billingiae (55.0)	2011	492,469	1,684	Headful packaging	43,866	43.8	70 (47.1)	Pseudomonas phage Epa40 (MT118304.1)	13	65.08
AH05	Pseudomonas syringae (58.8)	2011	886,451	3,301	Terminal repeats, 221 bp	40,502	57.2	51 (54.9)	Pseudomonas phage FRS (MZ598487.1)	95	94.72

a The complete genome of each phage was queried with BLASTn against the nucleotide database (nt) restricted to phages (taxid: 10699, 10662, and 10744). For each search, the best match to a complete genome is reported including query coverage, % identity, and accession number.

Each phage was single-plaque purified at least three times on its isolation host and amplified by overnight culturing in 10 ml King’s broth and 100 μl of the host (1). The cultured lysate was filtered (pore size, 0.45 μm), and following the kit protocol for the Promega Wizard PCR Preps DNA purification system (no. 7170), phage DNA was extracted by the Koskella lab. At North Carolina State University’s Genomic Science Laboratory, libraries for each DNA sample were prepared following the protocol for the Illumina TruSeq Nano DNA library prep kit and sequenced on the Illumina MiSeq platform, using a v3 150 SE flow cell. Genome assembly was performed at Gettysburg College, using the GS v2.9 *de novo* assembler (6). For each phage, 150-bp reads were assembled into one contig with >1,000× coverage and the contig consensus quality was verified using Consed v29 (6, 7) (Table 1). The genome ends were determined using PAUSE and PhageTerm (8, 9) (Table 1). The finished sequences were imported into DNA Master v5.22.22 (10) to map and compare the open reading frames. Putative genes were called based on both Glimmer v3.0 and GeneMark v2.5 algorithms (11, 12). Putative functions of the gene products were predicted using BLAST v2.12 (13) and HHpred (14). For the BLASTp matches, an E value below 10^−5^ was required to assign a function. For the HHpred matches, a high probability (>85%), substantial coverage (>50%), and low E value (<10^−5^) were required. The presence of tRNA genes was determined through the Web-based program ARAGORN (15). Default settings were used in all programs.

These phages have double-stranded DNA (dsDNA) genomes ranging from 39,095 to 46,062 bp and containing 51 to 70 protein coding genes (Table 1). Three phages—AH01, AH02, and AH03—have a genome organization typical of *Siphoviridae*, with structural genes showing a conserved order (16). Their assignment to this family is supported by BLASTn matches to *Siphoviridae* phages but with varying query coverage (Table 1). The best matches for AH02 and AH03 have low coverage; these two phages are substantially different from previously sequenced phages. Pseudomonas phage AH05 shows nucleotide similarity to *Podoviridae* Pseudomonas phages (Table 1). The GC contents of AH01, AH02, and AH05 are comparable to, if somewhat lower than, that of their isolation host (Table 1). In contrast, AH03 has a much lower GC content than its isolation host and also contains a tRNA gene for serine (anticodon gcu). Three other phage isolates—from two different leaves on the same tree and from a leaf on a second tree—were sequenced following the above protocols and determined to be identical to AH03.

### Data availability.

The genome sequences and associated information can be found under BioProject accession no. PRJNA754193 and GenBank/SRA accession no. MZ501269/SRX11736852 (AH01), MZ501271/SRX11736853 (AH02), MZ501266/SRX11736854 (AH03), and MZ501272/SRX11736856 (AH05).
